# Autophagy-Related Proteins Influence Mouse Epididymal Sperm Motility

**DOI:** 10.3390/ijms262411895

**Published:** 2025-12-10

**Authors:** Lorena Rodríguez-Páez, Jonathan J. Magaña, Charmina Aguirre-Alvarado, Verónica Alcántara-Farfán, Germán Chamorro-Cevallos, José Melesio Cristóbal-Luna, Erika Rosales-Cruz, Elba Reyes-Maldonado, Guadalupe Elizabeth Jiménez-Gutiérrez, Joaquín Cordero-Martínez

**Affiliations:** 1 Laboratorio de Bioquímica Farmacológica, Departamento de Bioquímica, Escuela Nacional de Ciencias Biológicas, Instituto Politécnico Nacional, Prol. Carpio y Plan de Ayala, Col. Santo Tomás, Mexico City 11340, Mexico; lrodrig@ipn.mx (L.R.-P.); caguirrea@ipn.mx (C.A.-A.); valcantaraf@ipn.mx (V.A.-F.); 2 Laboratorio de Medicina Genómica, Instituto Nacional de Rehabilitación Luis Guillermo Ibarra Ibarra, Mexico City 14389, Mexico; magana.jj@tec.mx; 3 Department of Bioengineering, Escuela de Ingeniería y Ciencias, Tecnologico de Monterrey, Campus Ciudad de México, Mexico City 14380, Mexico; 4 Unidad de Investigación Médica en Inmunología e Infectología, Centro Médico Nacional, La Raza, Instituto Mexicano del Seguro Social, Mexico City 02990, Mexico; 5 Laboratorio de Toxicología Preclínica, Departamento de Farmacia, Escuela Nacional de Ciencias Biológicas, Instituto Politécnico Nacional, Av. Wilfrido Massieu, Col. Zacatenco, Del. Gustavo A. Madero, Mexico City 07360, Mexico; jcristoball@ipn.mx (J.M.C.-L.); 6 Laboratorio de Investigación en Hematopatología, Departamento de Morfología, Escuela Nacional de Ciencias Biológicas, Mexico City 11340, Mexico; erosalesc@ipn.mx; 7 Laboratorio de Citología, Departamento de Morfología, Escuela Nacional de Ciencias Biológicas, Mexico City 11340, Mexico; elreyes@ipn.mx

**Keywords:** spermatozoa, motility, LC3, p62/SQSTM1, mTOR, acrosome reaction

## Abstract

Autophagy is an intracellular process that recycles and degrades cytoplasmic components, including organelles and macromolecules, to provide energy and basic components for cell survival, maintain cellular homeostasis, and avoid self-damage. It is currently not fully known if mouse sperm undergoes the autophagy process, nor is the subcellular distribution, protein levels of autophagy-related proteins, and the biological role of autophagy in epididymal mouse sperm physiology fully understood. We aimed to investigate key autophagy markers, including LC3 (microtubule-associated protein 1A/1B-light chain 3), p62/SQSTM1 (Sequestosome 1), and mTOR (mechanistic Target of Rapamycin), in epididymal mouse sperm under capacitation (Cap) or non-capacitation (NC) conditions. Furthermore, we evaluated the possible role of these autophagy-related proteins on sperm viability, motility, intracellular pH (pHi), intracellular calcium concentrations [Ca^2+^]i, mitochondrial membrane potential, and acrosome reaction (AR) induction in the presence or absence of chloroquine (CQ), K67, and rapamycin. Our results suggest a dynamic re-localization of the autophagy-related proteins LC3, p62/SQSTM1, and mTOR under capacitation conditions. Moreover, treatment with specific autophagy inhibitors, such as CQ and K67, resulted in decreased LC3-II and p62/SQSTM1 protein levels. Additionally, rapamycin did not increase mTOR levels. Interestingly, treatment with these inhibitors also resulted in decreased motility, reduced mitochondrial membrane potential and hindered AR induction without affecting sperm viability. Overall, the presence and dynamic re-localization of LC3, p62/SQSTM1, and mTOR suggest that mouse epididymal sperm could perform initial steps of autophagy under capacitation conditions, and results of the pharmacological treatment could be associated with an important role of these autophagy-related proteins in sperm motility and AR induction.

## 1. Introduction

Autophagy is an intracellular process that recycles and degrades cellular components, including organelles and macromolecules, to provide energy and basic components for cell survival and to overcome sublethal stress by activating signaling pathways for cellular homeostasis [1,2,3]. Autophagy occurs in mammalian sperm before their differentiation, playing a key role in different stages of spermatogenesis, including spermatogonial proliferation and differentiation, spermiogenesis, spermiation, and acrosome and flagellum biogenesis [4]. Furthermore, autophagy regulates spermatogenesis and male fertility, playing a cytoprotective role in preserving the survival of differentiating spermatogonia [5]. In recent years, the role of autophagy, as well as the function of the proteins involved in this process—such as microtubule-associated proteins 1A/1B light chain 3A (LC3), autophagy-related protein 5 (Atg5), autophagy-related protein 16 (Atg16), Beclin1, sequestosome-1 (p62/SQSTM1), mammalian target of rapamycin (mTOR), AMP-activated protein kinase 1/2 (AMPKα1/2), and PTEN-induced kinase 1 (PINK1)—has been elucidated in ejaculated human sperm [6]. LC3-I is processed to become LC3-II, one of the main components of the autophagosome membrane, which is important for its elongation and completion; it also transfers the cargo of this structure by interacting with p62/SQSTM1 [7]. Moreover, the main role of p62/SQSTM1, which co-localizes with LC3 in ejaculated human sperm [6], is recognizing ubiquitinated mitochondrial protein cargo and interacting with the autophagosome during boar sperm mitophagy after fertilization [8]. The mammalian target of rapamycin (mTOR), also known as the mechanistic target of rapamycin, has a major role in signaling pathways, including the coordination of cytoskeletal processes, cell survival, lipid synthesis, protein synthesis, and autophagy [9]. mTOR has two functional and structural forms, i.e., mTORC1 and 2; the difference lies in the proteins associated with the complex, mTORC1 being a rapamycin-sensitive complex [10]. The role of mTOR in mouse spermatogenesis has been reported; however, the mechanism underlying mTOR’s influence on spermatogonia proliferation and apoptosis is still under debate [11].

Despite our current knowledge of human ejaculated sperm autophagy, the possible presence and biological role of LC3, p62/SQSTM1, and mTOR in epididymal mouse sperm have not been described yet under capacitation and non-capacitation conditions. Interestingly, studies report that these proteins are modulated by intracellular pH (pHi) and Ca^2+^ flux in somatic cells [12,13,14]. Moreover, these capacitation-related mechanisms are essential for sperm viability and motility. However, how pHi and Ca^2+^ flux influence the autophagic process in mouse sperm—and the subsequent effects on reproduction—remains to be elucidated. The aim of this work is to analyze the presence and subcellular localization of LC3, p62/SQSTM1, and mTOR in epididymal mouse sperm in order to elucidate whether epididymal mouse sperm are able to perform the autophagy process under capacitation conditions. Furthermore, we evaluate the possible involvement of these autophagy-related proteins in the viability, motility, pHi, intracellular calcium concentration [Ca^2+^]i, acrosome reaction (AR) induction, and mitochondrial membrane potential of mouse epididymal sperm, associated with the treatment with autophagy inhibitors chloroquine (CQ), K67, and rapamycin.

## 2. Results

### 2.1. LC3 Is Expressed in Mouse Epididymal Sperm

**Figure 1:**
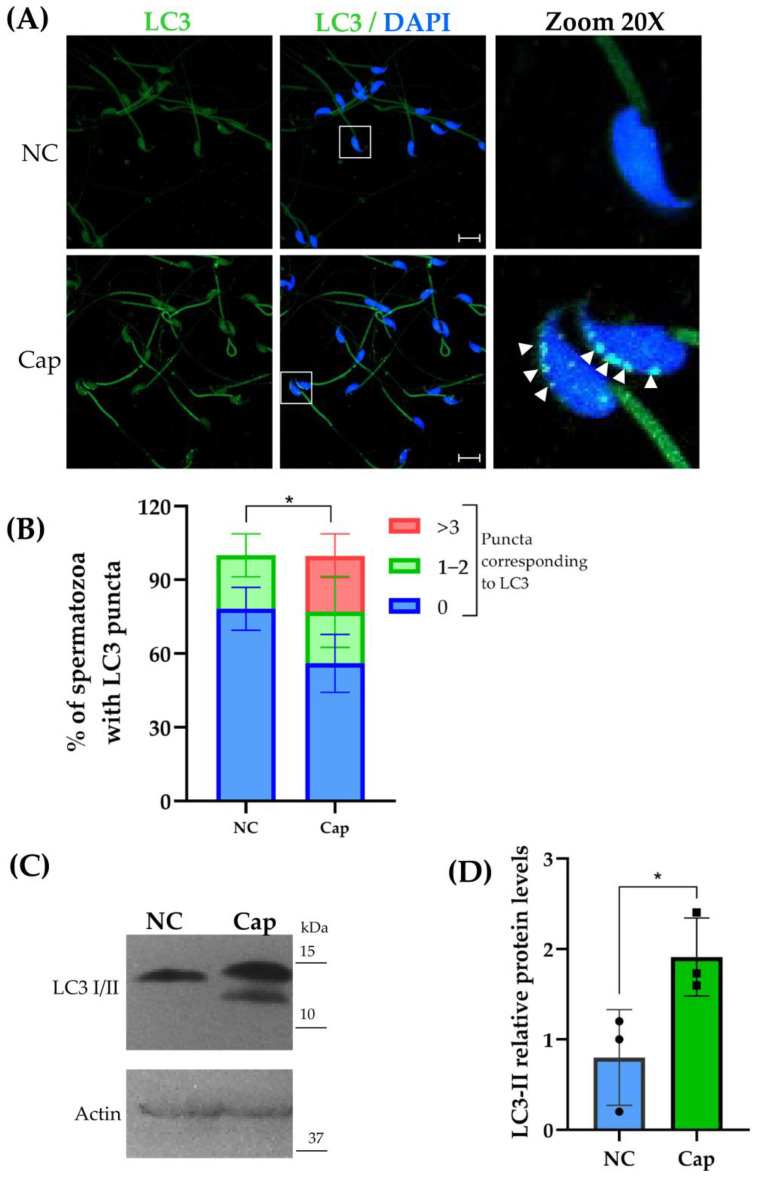
LC3 expression in mouse epididymal sperm under NC (non-capacitation) and Cap (capacitation) conditions. (**A**) Representative confocal laser scanning microscopy (CLSM) images of epididymal sperm immunostained for LC3. Nuclei were counterstained with DAPI. Scale bar = 50 μm. Squares represent the specific region magnified in the corresponding zoomed panel; arrows indicate LC3 redistribution in sperm (**B**) Percentage of sperm exhibiting LC3 puncta. Data represent mean ± SEM from three independent experimental replicates (*n* = 15 mice, 3 runs × 5 mice). (Fifty sperm per condition in each replicate were visualized. Statistical significance was assessed using Student’s *t*-test (* *p* < 0.05 vs. NC). (**C**) Representative Western blot of LC3 expression in total protein pooled sperm lysates, with Actin as a loading control. (**D**) Densitometric analysis of LC3 protein levels from Western blots. Data are presented as mean ± SEM from three independent experiments. Statistical significance was determined using Student’s *t*-test (*n* = 30 mice, 3 runs × 10 mice, * *p* < 0.05 vs. NC).

To analyze the subcellular localization and the possible role of autophagic proteins on mouse sperm, we evaluated LC3, one of the standard markers for autophagosomes. Interestingly, our results show a differential localization of LC3 under capacitation and non-capacitation conditions. On one hand, LC3 appears homogeneously located in the head and the principal piece of the sperm flagellum under non-capacitation conditions. On the other hand, under capacitation conditions, LC3 changes its localization to form puncta around the sperm head; this distribution is consistent and suggests autophagosome formation. This pattern shows greater intensity in the sperm head, suggesting the presence of distinct protein pools across the acrosome region (Figure 1A). Additionally, the number of puncta significantly increases in sperm under capacitation conditions (Figure 1A). Consistent with these observations, the LC3 protein levels were also evaluated, and the results show a higher proportion of the LC3-II/I ratio on capacitated compared to non-capacitated mouse epididymal sperm (Figure 1B). These results suggest that the autophagy process is present in mouse epididymal sperm under capacitation conditions.

### 2.2. p62/SQSTM1 Decreases Under Capacitation Conditions in Mouse Epididymal Sperm

**Figure 2:**
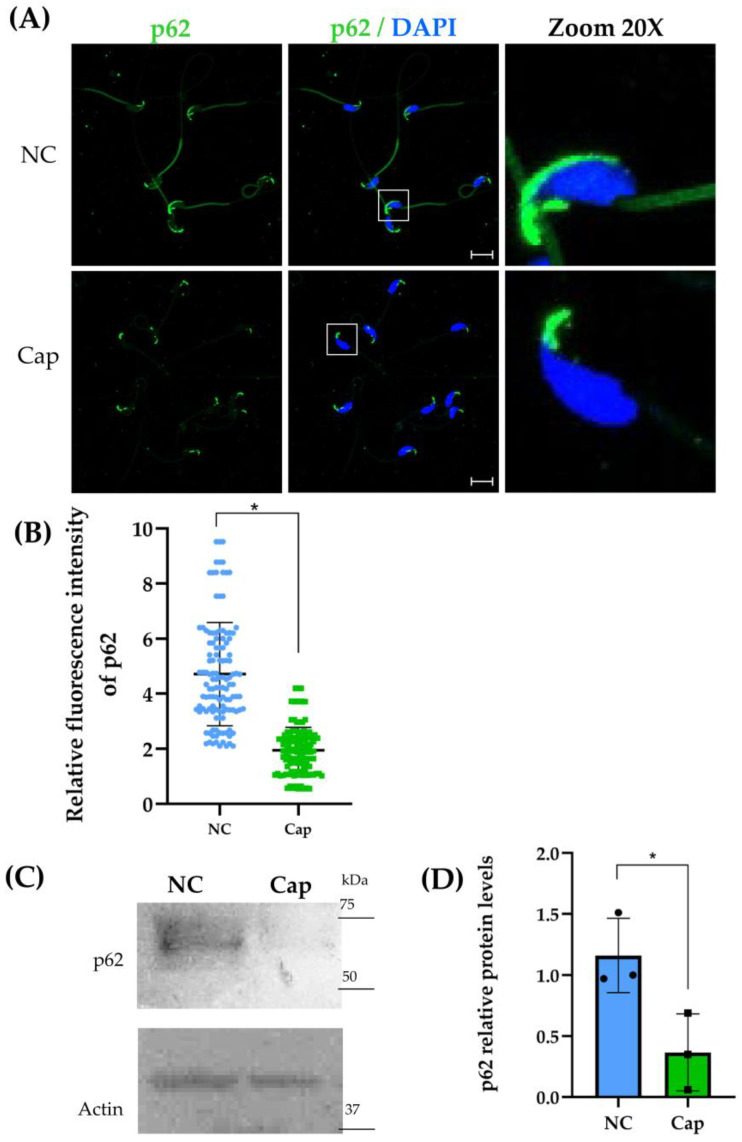
p62/SQSTM1 expression in mouse epididymal sperm under NC (non-capacitation) and Cap (capacitation) conditions. (**A**) Representative confocal laser scanning microscopy (CLSM) images of epididymal sperm immunostained for p62/SQSTM1. Nuclei were counterstained with DAPI. Scale bar = 50 μm. Squares represent the specific region magnified in the corresponding zoomed panel. (**B**) Relative fluorescence intensity. Data represent mean ± SEM from three independent experimental replicates (n = 15 mice, 3 runs × 5 mice). (Fifty sperm per condition in each replicate were visualized. Statistical significance was assessed using Student’s *t*-test (* *p* ≤ 0.05 vs. NC). (**C**) Representative Western blot of p62/SQSTM1 expression in total protein pooled sperm lysates, with Actin as a loading control. (**D**) Densitometric analysis of p62/SQSTM1 protein levels from Western blots. Data are presented as mean ± SEM from three independent experiments. Statistical significance was determined using Student’s *t*-test (n = 30 mice, 3 runs × 10 mice, * *p* < 0.05 vs. NC).

LC3 is a main marker for autophagosome formation and a significant partner for other autophagic proteins, such as p62/SQSTM1. Therefore, we evaluated its presence and subcellular distribution. Our results showed the presence of p62/SQSTM1 in mouse epididymal sperm, mainly located in the acrosome region, but also in the head and the middle piece of the sperm flagellum under non-capacitation conditions (Figure 2A). Moreover, under capacitation conditions, the fluorescence intensity decreased drastically in the principal piece of the sperm flagellum and in the acrosome region of the sperm head (Figure 2A). Furthermore, the p62/SQSTM1 protein level significantly decreased under capacitation conditions (Figure 2B). These results also suggest that the autophagy process is present in mouse epididymal sperm under capacitation conditions.

### 2.3. mTOR Is Expressed in Mouse Epididymal Sperm

**Figure 3:**
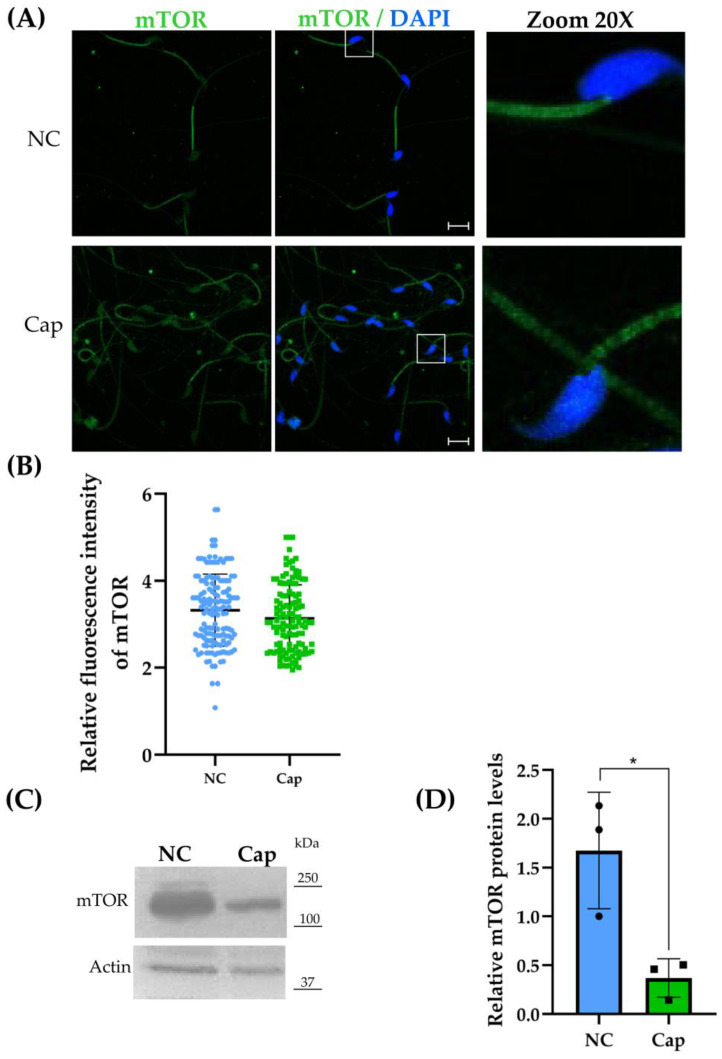
mTOR expression in mouse epididymal sperm under NC (non-capacitation) and Cap (capacitation) conditions. (**A**) Representative confocal laser scanning microscopy (CLSM) images of epididymal sperm immunostained for mTOR. Nuclei were counterstained with DAPI. Scale bar = 50 μm. Squares represent the specific region magnified in the corresponding zoomed panel. (**B**) Relative fluorescence intensity. Data represent mean ± SEM from three independent experimental replicates (*n* = 15 mice, 3 runs × 5 mice). Fifty sperm per condition in each replicate were visualized. Statistical significance was assessed using Student’s *t*-test (* *p* = 0.2318 vs. NC). (**C**) Representative Western blot of mTOR expression in total protein pooled sperm lysates, with Actin as a loading control. (**D**) Densitometric analysis of mTOR protein levels from Western blots. Data are presented as mean ± SEM from three independent experiments. Statistical significance was determined using Student’s *t*-test (*n* = 30 mice, 3 runs × 10 mice, * *p* < 0.05 vs. NC).

One critical regulator of autophagy is mTOR, a central sensor that integrates different signals in anabolism for biosynthetic and bioenergetic requirements. Sperm were incubated under non-capacitation and capacitation conditions to observe the presence and subcellular localization of mTOR. Our results are consistent with the presence of mTOR, mainly in the middle piece of the flagellum, and a homogeneous fluorescence in the sperm head (Figure 3A) under non-capacitation conditions. Under capacitation conditions, the fluorescence intensity signal in the middle piece of the flagellum decreased, although not significantly (Figure 3A). Furthermore, the protein level of mTOR considerably decreased under capacitation conditions compared to non-capacitation conditions (Figure 3B). Low levels of mTOR in mouse epididymal sperm under capacitation conditions suggest the existence of the autophagy process.

### 2.4. Effects of Exposure to Different CQ, K67, and Rapamycin Concentrations on Mouse Sperm Viability

**Figure 4:**
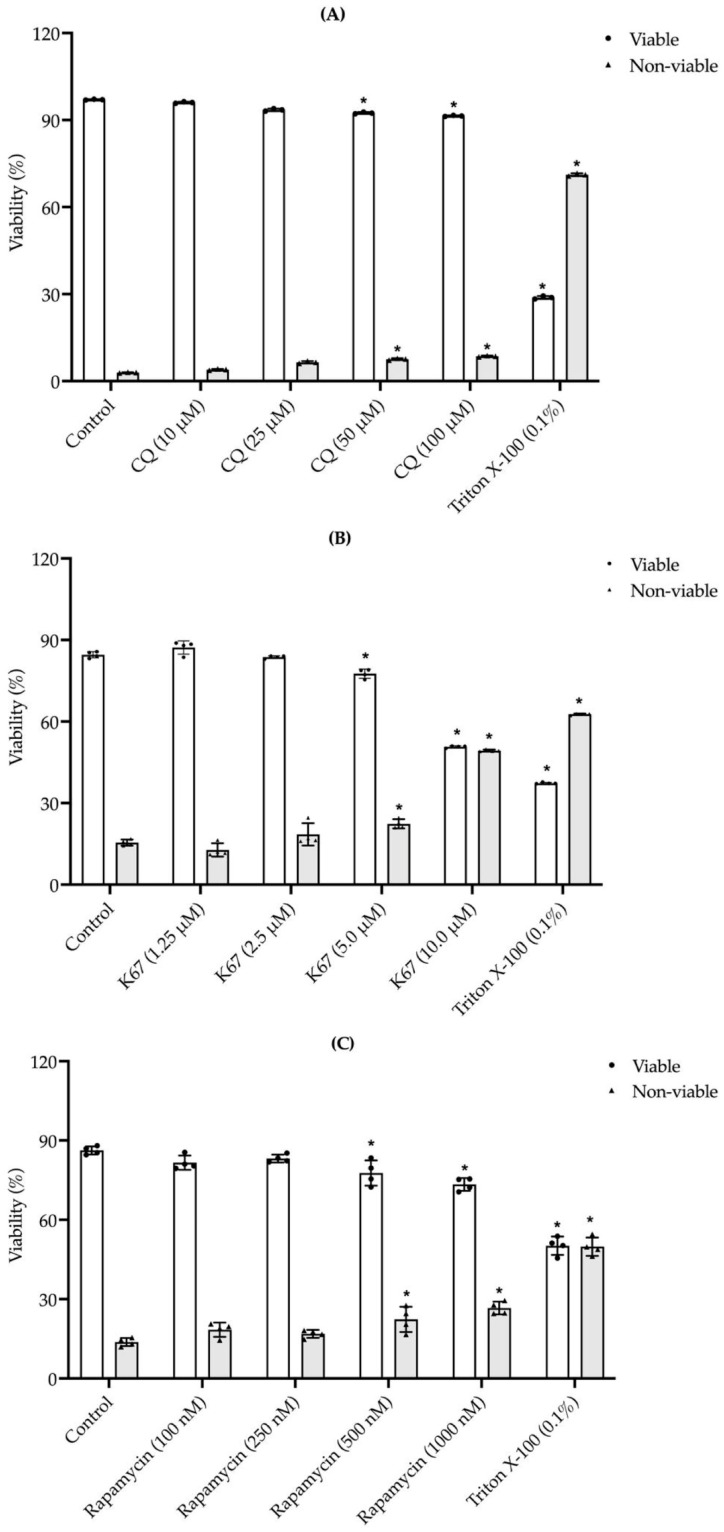
Effects of CQ, K67, and rapamycin exposure on mouse sperm viability. The graphs show the effects of (**A**) CQ, (**B**) K67, and (**C**) rapamycin on epididymal mouse sperm after 90 min of capacitation. Concentrations: 25 mM, 2.5 mM, and 250 nM, respectively. Data are presented as mean ± SEM (*n* = 15 mice, 3 runs × 5 mice). Asterisks indicate significant differences (* *p* < 0.05 vs. control). Statistical analyses were conducted using the Brown–Forsythe test, and comparisons were made using one-way ANOVA and post hoc Dunnett test.

The evaluation of a possible toxic effect showed that lower CQ (10 and 25 μM), K67 (1.25 and 2.5 μM), and rapamycin (100 and 250 nM) (Figure 4A–C) concentrations did not affect sperm viability; however, high CQ (50 and 100 μM), K67 (5 and 10 μM), and rapamycin (500 and 1000 nM) concentrations decreased sperm viability in a statistically significant manner (Figure 4A–C). According to these results and the selected concentrations used in previous reports [6], we selected CQ (25 μM), K67 (2.5 μM), and rapamycin (250 nM) for further experiments.

### 2.5. Effects of CQ, K67, and Rapamycin on LC3, p62/SQSTM1, and mTOR Protein Levels

**Figure 5:**
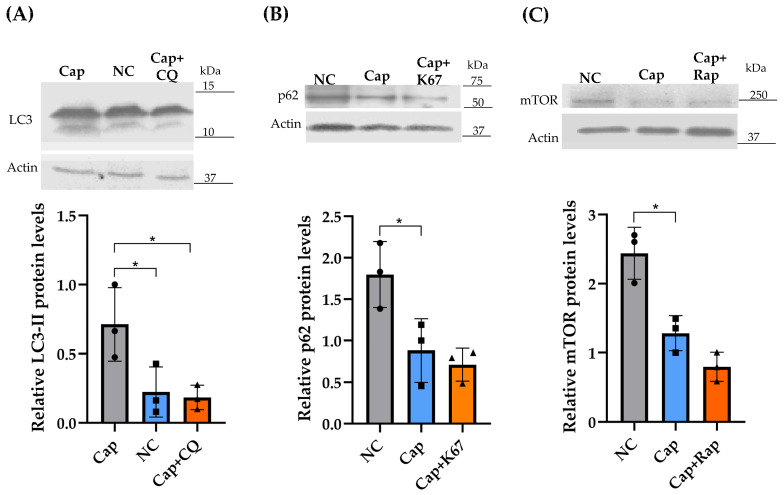
Effects of CQ, K67, and rapamycin on LC3, p62/SQSTM1, and mTOR protein levels. The graphs show the effect of CQ on LC3 levels (**A**), the effect of K67 on p62/SQSTM1 levels (**B**), and the effect of rapamycin on mTOR levels under capacitation conditions (Cap) (**C**). Data are presented as mean ± SEM. Asterisks indicate significant differences (*n* = 30 mice, 3 runs × 10 mice) (* *p* < 0.05 vs. Cap). Statistical analyses were performed using the Brown–Forsythe test followed by Bartlett’s test, and comparisons were performed using a one-way ANOVA test and post hoc Dunnett test.

Considering our previous findings on the dynamic subcellular localization of autophagy-related proteins, such as LC3, p62, and mTOR, under non-capacitation and capacitation conditions on epididymal mouse sperm, we evaluated the effects of different autophagy inhibitors. Specifically, we aimed to analyze their potential effects under capacitation and non-capacitation conditions. To this end, we used concentrations of the CQ, K67, and rapamycin inhibitors based on dose–effect assays (Figure 4), identifying concentrations that did not alter sperm viability. Under capacitation conditions, LC3-II protein levels decreased in the CQ-treated samples compared to the corresponding capacitation control sample (Figure 5A). Although p62/SQSTM1 protein levels were slightly reduced in the capacitated samples treated with the K67 inhibitor relative to the capacitation control sample (Figure 5B), the differences were not statistically significant. Likewise, mTOR protein levels in the presence of rapamycin during capacitation slightly decreased in a similar fashion compared with their respective capacitation control (Figure 5C). As a negative control, the levels of these autophagy-related proteins were also assessed under non-capacitation conditions (Figure 5A–C).

### 2.6. The Presence of CQ, K67, and Rapamycin Reduces Mouse Sperm Motility

**Figure 6:**
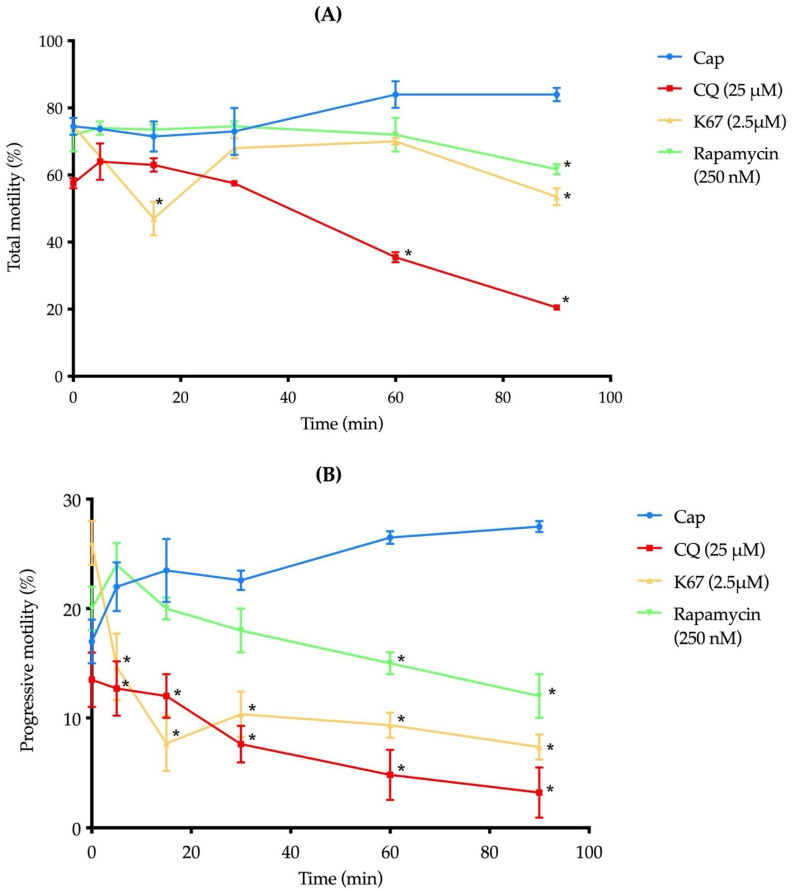
Effects of CQ, K67, and rapamycin on mouse sperm motility. The effects of CQ (25 µM), K67 (2.5 µM), and rapamycin (250 nM) on mouse sperm total motility (**A**) and progressive motility (**B**) after 90 min of incubation were evaluated under capacitation conditions (Cap). Data are presented as mean ± SEM (*n* = 15 mice, 3 runs × 5 mice). Asterisks indicate significant differences (* *p* < 0.05 vs. Cap). Statistical analyses were performed using the Brown–Forsythe test, and group comparisons were conducted using one-way ANOVA and post hoc Dunnett test.

To fertilize an oocyte, mammalian spermatozoa must travel through the female tract. This ability, known as motility, is considered the fundamental requirement of sperm viability and fertility [15]. In this context, we evaluated the effect of the previously described autophagy inhibitors on mouse sperm motility under capacitation conditions. Sperm treated with CQ and rapamycin showed decreased motility 60 min after the incubation started. In contrast, sperm treated with K67 showed a significant reduction in motility after 15 min of incubation. Our results indicate that all the inhibitors reduced motility at 90 min compared to the control samples (Figure 6A). Additionally, CQ and K67 reduced progressive motility from 5 min to the end of the incubation (Figure 6B). Rapamycin also reduced the progressive motility from 60 min to the end of the incubation period (Figure 6B).

### 2.7. CQ, K67, and Rapamycin Do Not Induce Changes in Intracellular Calcium Concentrations [Ca^2+^]i

**Figure 7:**
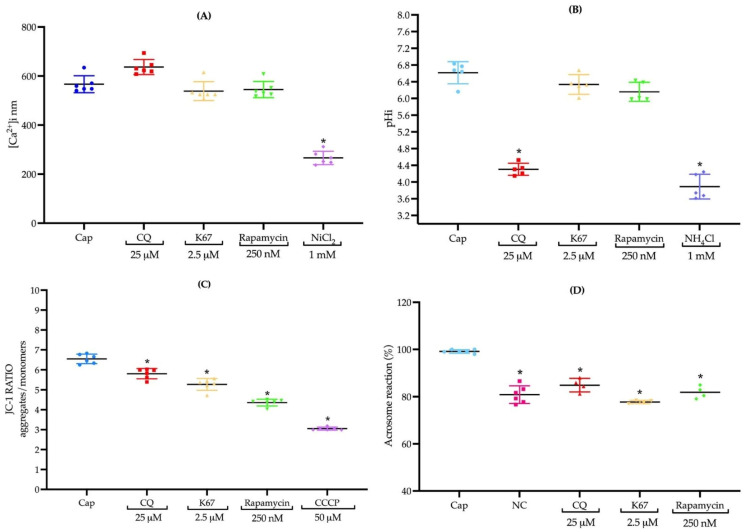
Effects of CQ, K67, and rapamycin on mouse sperm intracellular calcium concentration ([Ca^2+^]_i_), intracellular pH (pH_i_), membrane potential, and the acrosomal reaction (AR). (**A**) Effects of CQ (25 μM), K67 (2.5 mμM), and rapamycin (250 nM) on [Ca^2+^]_i_ levels after 90 min of incubation under capacitation conditions. (**B**) Effects of the same treatments on pH_i_. (**C**) Membrane potential changes following exposure to CQ, K67, and rapamycin. (**D**) The acrosomal reaction (AR) after treatment with CQ, K67, and rapamycin. Data are presented as median or mean ± SEM (*n* = 15 mice, 3 runs × 5 mice). Asterisks indicate significant differences (* *p* < 0.05 vs. Cap). Statistical analyses were conducted using the Brown–Forsythe test, and group comparisons were performed using one-way ANOVA and post hoc Dunnett test.

Considering the close relationship between intracellular calcium, spermatic properties, and autophagy, we evaluated whether treatments with autophagy inhibitors would indicate a role in sperm viability and motility. We analyzed [Ca^2+^]i under capacitation conditions, but none of the drugs induced any change in [Ca^2+^]i after 90 min of incubation (Figure 5A). Nevertheless, CQ treatment induced a trend of increasing [Ca^2+^]i but without statistical significance (Figure 7A).

### 2.8. Inhibitors Do Not Influence pHi, Except CQ

Intracellular calcium and pHi are interconnected, both playing pivotal roles in sperm motility, capacitation [16,17], and autophagy flux. Given this relationship and our observation that intracellular calcium levels remain unchanged after inhibitor treatment, we evaluated whether pHi fluctuates in response to various autophagy inhibitors. Our results revealed that only CQ induced drastic acidification in pHi (Figure 7B). 

### 2.9. The Presence of CQ, K67, and Rapamycin Inhibits Mitochondrial Membrane Potential

Another crucial process for both sperm capacitation and autophagy is mitochondrial activity. Therefore, we evaluated mitochondrial membrane potential and assessed the possible effects after treatment with our inhibitors. The results showed that all the drugs inhibited mitochondrial membrane potential in a statistically significant manner (Figure 7C). The inhibition of mitochondrial membrane potential suggests that the presence of CQ, K67, and rapamycin blocks sperm mitophagy. The effect was validated by using CCCP 50 μM as a negative control (Figure 7C).

### 2.10. Treatments with CQ, K67, and Rapamycin Impede AR Induction in Mouse Sperm

Once we determined a possible inhibition of sperm mitophagy, we evaluated AR induction in the presence of the three drugs. Our results showed that the presence of CQ, K67, and rapamycin impeded AR induction in a statistically significant manner (Figure 7D). This effect was validated by using a sample incubated under non-capacitation conditions as a control (Figure 7D).

## 3. Discussion

Autophagy plays a central role in cellular homeostasis, contributing to cell survival, development, and differentiation; despite its importance, studies on the involvement of autophagy in sperm function remain unexplored [18]. The aim of our work is to identify and analyze autophagy markers and their probable function in sperm under capacitation and non-capacitation conditions. Among the proteins involved in autophagy, LC3 plays an important role in autophagosome formation and maturation and also works as an adaptor protein to recruit selective cargo to the autophagosome by interacting with cargo receptors [19,20]. As one of the main autophagy markers, LC3 showed a spatial redistribution under capacitation conditions, being redistributed to regions overlapping with mitochondria, suggesting the possible presence of LC3 inside the mitochondrial sheath [21], and mostly in the apical acrosome region, forming puncta (Figure 1A) [22], which is consistent with a possible co-localization with acrosomal markers as Peanut agglutinin (PNA) or Pisum sativum agglutinin (PSA) [23]. The appearance of LC3 puncta is also consistent with the possible autophagosome formation and the initiation of the autophagy process. In freshly ejaculated human sperm, LC3 showed a similar subcellular localization to that observed in mouse epididymal sperm under capacitation conditions; nevertheless, after 2 h of incubation under capacitation conditions, human sperm appear to show the same LC3 protein aggregates, only in the middle piece of the flagellum (Figure 1A) [6]. Moreover, under capacitation conditions, LC3-II significantly increases, suggesting that in epididymal mouse sperm, the autophagy process is probably induced or reduced (Figure 1C,D). However, we can argue that the difference between our results and ejaculated human sperm data is due to the presence of various molecules in the seminal fluid [24], which makes these spermatozoa behave differently during the induction of capacitation or the acrosome reaction.

p62/SQSTM1 is a key multidomain protein with various functions, among which we found the disposal of poly-ubiquinated aggregates via lysosomes, the removal of different cargo types (bacteria, viruses, and organelles) through its role as a receptor, and a binding site to interact with LC3 [25]. It is important to mention that p62/SQSTM1 is degraded during the autophagy process; therefore, a loss of the fluorescence signal of p62/SQSTM1 after capacitation (Figure 2A) and a decrease in protein levels (Figure 2B) suggest that sperm could be experiencing autophagy [16]. Both LC3-p62/SQSTM1 are altered under capacitation conditions (Figure 1 and Figure 2); however, in ejaculated horse spermatozoa during AR induction, LC3 undergoes a light redistribution into acrosomal membranes [7], suggesting a continuation of autophagy to further steps given by an increment in the processing of LC3-I into LC3-II. We can argue that the redistribution of LC3 forming puncta and changes in the fluorescence signal of p62/SQSTM1 into acrosome membranes are due to the phospholipid modification necessary for autophagosome formation performed by this lysosome-related organelle derived from the Golgi apparatus [17,18].

mTOR is a Ser/Thr protein kinase present in all mammalian and non-mammalian cells, which modulates protein synthesis, proliferation, survival, motility, and even autophagy. Recently, mTOR was found to be associated with spermatogenesis via controlling nutritional support [10,26]. mTOR has two functional and structural forms, i.e., mTORC1 and 2; the difference lies in the proteins associated with the complex. In addition, mTORC1 is a rapamycin-sensitive complex [10]. mTORC1 negatively regulates autophagy by activating the autophagy suppressor DAP1, regulating TFEB, and preventing lysosome biogenesis [9]. The reduced fluorescence intensity in the middle piece of the sperm flagellum (Figure 3A), as well as the reduced mTORC1 levels under capacitation conditions, is consistent with an attenuation of mTORC1 repression, which in turn would allow autophagy to begin (Figure 3C,D) [27]. Currently, there is no report on the subcellular localization of mTOR in mature sperm from rodents; however, in freshly human ejaculated sperm, its presence has been described only by Western blot analysis [6].

CQ is a weak base that induces increased pH in cellular compartments, inhibits hydrolase activity, and decreases autophagosome–lysosome formation mediated by LC3 [28,29]. Previous works reported the use of CQ in higher concentrations (50 μM) [6] than we use in this work (25 μM), ensuring that CQ did not affect sperm viability (Figure 5A). The treatment with CQ considerably reduced LC3-II protein levels under capacitation conditions compared to the control sample (Figure 4A). These results suggest an early impairment in autophagosome formation or maturation in the autophagy process [30]. In contrast, in human ejaculated sperm, the presence of CQ 50 μM induced an accumulation of LC3-II [6], suggesting late impairment in the fusion and degradation steps [30]. Previous reports have demonstrated that CQ (250 μg/mL) induces a reduction in human sperm motility [31], which is consistent with our findings, where CQ drastically reduced total and progressive motility (Figure 6A,B). Capacitation-related parameters, such as [Ca^2+^]i and pHi, were also evaluated, where CQ treatment resulted in a slight but not statistically significant increase in [Ca^2+^]i (Figure 7A). However, CQ promoted a drastic decrease in pHi (Figure 7B). In previous works on freshly ejaculated human sperm, CQ induced a decrease in [Ca^2+^]i with no effects on pHi [6]. Unprotonated form of CQ can freely diffuse across acidic endolysosomes and Golgi membranes, then CQ is protonated and accumulated, neutralizing the acidic pH of these organelles; moreover, after 1 hr of treatment, CQ acidifies the cytosol of cancer cells [32,33]. Additionally, the presence of CQ impeded AR induction in a statistically significant manner (Figure 7D).

We can hypothesize that failure of AR induction in presence of CQ probably correlates with the decrease in LC3-II protein level (as also observed in the presence of this drug under capacitation conditions (Figure 5A) or is a direct effect of CQ on mitochondrial membrane potential (Figure 7C), which is consistent with previous findings, where CQ induced the collapse of mitochondrial membrane potential in human heart mitochondria [34].

K67 inhibits the interaction between Kelch-like ECH-associated protein 1 (KEAP1) and both nuclear factor erythroid 2-related factor 2 (NRF2)-ETGE and phosphorylated-p62; therefore, K67 blocks the autophagy process [35]. Recent evidence has shown the cytotoxic effect of K67 against the A549 cancer lung cell line, where 10 μM of this drug did not affect cell viability even after 48 h of incubation [36]. This evidence supports our observation that sperm viability was not affected after treating the sperm suspension with 2.5 μM K67 (Figure 5B). To date, no study has evaluated p62/SQSTM1 protein levels in the presence of K67 or another inhibitor, either in human ejaculated or mouse epididymal sperm. In contrast, our results showed a decrease in p62/SQSTM1 levels in the presence of K67 compared to the control sample (Figure 4B). This reduction may not reflect the activation of autophagic flux; rather, it is consistent with a specific effect of K67, as previous studies have reported that K67 can lead to a significant decrease in phospho-mimetic p62 protein levels by promoting Nrf2 degradation [35]. Similarly, in male mice, deltamethrin administration caused testicular damage, accompanied by a decrease in p62/SQSTM1 protein levels, enhancing autophagy flux and contributing to male infertility [37]. Thus, reduced p62/SQSTM1 protein levels may reflect an increase in sperm mitophagy activity, promoting an alteration in mitochondrial membrane potential [3]. Additionally, the K67 treatment decreased total and progressive motility (Figure 6A,B), without affecting [Ca^2+^]i and pHi (Figure 7A,B); nonetheless, AR induction was impeded by K67 (Figure 7D). No study has investigated the inhibition of p62/SQSTM1 by K67 in sperm from mammals; however, the presence of p62/SQSTM1 in the acrosome region and the middle piece of the sperm flagellum (Figure 2A) and the low-level fluorescence intensity in the principal piece of the sperm flagellum and in the acrosome region of the sperm head (Figure 2A) under capacitation conditions suggest a main role of p62/SQSTM1 in AR acquisition. Also, the presence of K67 induced a reduction in mitochondrial membrane potential (Figure 7C), which could be explained by p62/SQSTM1′s important role as a regulator of mitochondrial respiratory capacity, as previously reported in human cortical neurons [38].

Early reports showed that the use of rapamycin (500 nM) on human sperm did not affect cell viability after 2 h of incubation [6]. In our experiments, we assayed lower concentrations (250 nM) than those previously reported, and such concentrations did not affect sperm viability in a significant manner (Figure 4C). Therefore, the effect of the drug is also related to its pharmacological effect instead of its toxicity. Under physiological conditions, mTOR suppresses autophagy and, conversely, its inhibition should promote autophagy [39,40]; however, the presence of rapamycin did not induce a statistically significant increase in mTOR protein levels compared to the control sample (Figure 5C). We can hypothesize that this slight increase in mTOR suggests a modest inhibition of autophagy flux [9]; however, more evidence is needed to confirm this hypothesis. Treatment with rapamycin reduced both total and progressive motility after 90 and 60 min of incubation, respectively (Figure 6A,B). Recently, in human ejaculated sperm, it was found that the presence of rapamycin did not affect sperm motility [6]; however, our results are supported by previous reports where chronic administration of this drug caused side effects against mouse male fertility, including spermatogenic arrest and low motility [41]. On the other hand, rapamycin, as well as K67, did not affect [Ca^2+^]i or pHi (Figure 7A,B). Similar to CQ and K67, rapamycin impeded the AR induction. Conversely, previous works showed that the presence of rapamycin increased progressive and rapid motility of human freshly ejaculated sperm [6], with no effects on [Ca^2+^]i and pHi. This result suggests that the autophagy process is not related to capacitation or even that capacitation conditions do not support the progression of autophagy to further steps. Our calcium assay results (Figure 7A) support this suggestion, since calcium homeostasis plays a pivotal role in regulating mitophagy in numerous neurodegenerative diseases, where [Ca^2+^]i disruptions would lead to mitochondrial dysfunction [42]. mTOR is a key regulator of mitochondrial function, and its inhibition also produces alterations in mitochondrial membrane potential [43]. Accordingly, our results showed that rapamycin decreases mitochondrial membrane potential in a statistically significant manner (Figure 7C), which is consistent with previous findings where rapamycin alone reduced mitochondrial membrane potential in human glioblastoma cells [44]. Finally, all the inhibitors decreased total and progressive motility; therefore, we can argue that the inhibition of autophagy promotes the accumulation of recycling materials and the absence of progression in the autophagy process, producing cellular dysfunction as the impairment of mitochondrial membrane potential (Figure 7C) and blocking AR induction (Figure 7D). Moreover, failure of AR induction in the presence of the autophagy inhibitors (Figure 7D) suggests an active role of this lysosome-related organelle in autophagy. For example, AR induction in horse spermatozoa resulted in a significant increase in LC3 processing compared to capacitation conditions, which decreased AR in the presence of 3-methyladenine, suggesting that some components of the autophagy mechanism induce AR [7].

## 4. Materials and Methods

### 4.1. Materials and Reagents

Reagents were purchased from Mallinckrodt Baker (Phillipsburg, NJ, USA) or Sigma Chemical Co. (St. Louis, MO, USA), unless otherwise mentioned.

### 4.2. Antibodies

The primary antibodies included the following: mouse monoclonal antibodies against beta Actin Antibody ((C4) sc-47778; Santa Cruz Biotechnology, Dallas, TX, USA); rabbit polyclonal antibodies against mTOR (#2972s; Cell Signaling Technology, Danvers, MA, USA) and LC3 A/B (#4108; Cell Signaling Technology, MA, USA); and rabbit monoclonal antibodies against SQSTM1/p62 ((D6M5X) #23214; Cell Signaling Technology, MA, USA). HRP secondary antibodies were also used, including Goat Anti-Rabbit IgG H&L (HRP) (Ab6721; Abcam, Cambridge, UK) and Rabbit Anti-Mouse IgG H&L (HRP) (Ab6728; Abcam, Cambridge, UK). The fluorochrome-conjugated secondary antibody was Goat Anti-Rabbit IgG Antibody (H+L). Fluorescein (FI-1000; Vector Laboratories, Newark, CA, USA) was also used.

### 4.3. Animal Procedures

Adult male CD1 mice between 12 and 24 weeks old were obtained from the vivarium at Escuela Nacional de Ciencias Biológicas. They weighed between 20 and 25 g and were housed for a minimum of 3 weeks in polypropylene cages at room temperature. The mice had ad libitum access to standard commercial rodent food and water, following a regular light/dark cycle (Figure S1). The total number of specimens for the immunofluorescence and functional sperm parameter methods was 15 mice per condition (5 mice in each of the 3 experimental runs). For Western blot (WB) analysis, the total number of specimens used was 30 mice per condition (10 mice in each of the 3 experimental runs). In each experimental run, new specimens were used.

### 4.4. Sperm Sample Preparation

Epididymal sperm were collected via puncture using a physiological saline solution (154 mM) at 37 °C. Collections from five male mice (or ten specimens, depending on the experiment) were performed at 2 min intervals to obtain pooled sperm samples (aliquots) and were divided for non-capacitation and capacitation conditions in each independent experiment. The pooled sperm samples for each condition were centrifuged at 500× *g* for 6 min at room temperature and then resuspended in a capacitation Tyrode’s modified medium. This medium consisted of 120 mM NaCl, 2.8 mM KCl, 11.9 mM NaHCO_3_, 0.36 mM NaH_2_PO_4_, 0.49 mM MgCl_2_, 0.25 mM sodium pyruvate, 20 mM sodium lactate, 1.8 mM CaCl_2_, 5.56 μM glucose, and 1 mg/mL albumin at pH 7.6. A non-capacitation medium was prepared using the same Tyrode’s modified medium, but glucose and albumin were excluded, and 11.9 mM NaHCO_3_^−^ was substituted with 25 mM HEPES. Each experimental setup utilized new pooled sperm samples from 5 or 10 male mice (Immunofluorescence/sperm functional parameters and WB analysis, respectively) from the same day, to achieve a final concentration of 8–10 × 10^6^ spermatozoa per milliliter [45].

### 4.5. Immunofluorescence Assays

Sperm pooled samples were incubated under capacitation or non-capacitation conditions for 120 min at 37 °C. After incubation time, the sperm samples were immediately placed on gelatin-coated coverslips and fixed with 4% paraformaldehyde in PBS for 10 min. They were then permeabilized with 0.2% Triton X-100-PBS for 12 min and blocked with 2% bovine serum albumin (BSA) for 20 min. The coverslips were incubated overnight with the respective primary antibodies at 4 °C. The following day, the cells were washed twice with PBS before incubation with fluorochrome secondary antibodies for 1 h at room temperature. Finally, the coverslips were mounted with Vectashield/DAPI (Vector Laboratories, Newark, CA, USA) and further analyzed with confocal laser scanning microscopy (ZEISS LSM 900; Carl Zeiss Microscopy Jena, Germany) using a 63× (NA = 1.2) oil-immersion objective. Image analysis was performed using Image J software 1.54K, NIH, USA.

### 4.6. Western Blotting

Sperm pooled samples were incubated under capacitation or non-capacitation conditions for 120 min at 37 °C. Then, the total protein sperm lysates were immediately obtained with 75 μL from a lysis buffer (10 mM Tris-HCl, pH 8, 30 mM NaCl, 0.2% Triton X-100, 50 mM NaF, 1 mM PMSF, 1X complete protease inhibitor), sonicated three times at 5.5 mV with 10 s bursts, and centrifuged at 2000 rpm for 2 min at 4 °C. The supernatants were recovered, and their total protein concentration was quantified using a BCA colorimetric assay according to the manufacturer’s instructions (DC™ Protein Assay Kit II #5000112; Bio-Rad Laboratories Inc., Berkeley, CA, USA). A total of 150 µg of total protein sperm lysates were loaded per well for each experimental condition and electrophoresed on 8%, 10%, or 15% SDS–polyacrylamide gels (to analyze mTOR, p62, and LC3 protein levels, respectively) and transferred to Immobilon PVDF membranes (cat. IPVH00010 Millipore; Merck, Darmstadt, Germany). The membranes were blocked with 5% low-fat dried milk in TBST (100 mM Tris-HCL, pH 8.0, 150 mM NaCl, 0.5% (*v*/*v*) Tween-20) and incubated with the primary antibodies overnight at 4 °C. The protein signal was developed using the corresponding secondary antibodies and enhanced with luminol-based chemiluminescence from an ECL Substrate Kit (ECL Substrate Kit (High Sensitivity) ab133406; Abcam, Cambridge, UK). Images were acquired for densitometric analysis using the Gel Doc EX system (Bio-Rad Laboratories Inc., Berkeley, CA, USA) and analyzed with Image Lab 6.0.1 software (Bio-Rad Laboratories Inc., Berkeley, CA, USA). Fixed point normalization was performed to determine relative protein levels. Specifically, the raw densitometric intensity values of the control samples were normalized.

### 4.7. Evaluation of Mouse Sperm Viability in the Presence of CQ, K67, and Rapamycin

Sperm suspensions were incubated for 90 min in capacitation Tyrode’s media at pH 7.6 and 37 °C, either with or without different concentrations of CQ (10, 25, 50, and 100 μM), K67 (1.25, 2.5, 5, and 10 μM), and rapamycin (100, 250, 500, and 1000 nM), in dose-dependent curves. The concentration of each drug was selected according to previous reports [6]. Following the incubation, sperm viability was assessed using a double staining with fluorescein diacetate (FDA) and propidium iodide (PI). Flow cytometry analysis was conducted using a FACSAria cell sorter (Becton Dickinson, Towson, MD, USA) equipped with a 488 nm laser beam, and the data were processed with FACSDiva software version 6.1.3 (Becton Dickinson). To ascertain any potential adverse effects of the drugs on sperm viability, 0.1% Triton X-100 served as a negative control [46].

### 4.8. Sperm Treatments with the Autophagy Inhibitors CQ, K67, and Rapamycin

Sperm suspensions were independently incubated under capacitation conditions, non-capacitation conditions, as well as in the presence of CQ (25 μM), K67 (2.5 μM), and rapamycin (250 nM) under capacitation conditions for 120 min at 37 °C. Subsequent Western Blot analyses were performed immediately after, as described in Section 4.6.

### 4.9. Evaluation of Mouse Sperm Motility in the Presence of CQ, K67, and Rapamycin

In order to assess the impact of the different drugs on sperm motility, we incubated sperm suspensions with or without CQ 25 μM, K67 2.5 μM, and rapamycin 250 nM for 90 min. Throughout the incubation period, we sampled the sperm suspension at 0, 5, 15, 30, 60 and 90 min to analyze sperm motility quantitative parameters, specifically total and progressive motility, using a computer-assisted sperm analysis (CASA) system (TOX IVOS, software version 12.3, Hamilton-Thorne Biosciences, Beverly, MA, USA). The analysis was performed in a sample-counting chamber maintained at 37 °C (MicroCell 20 Micron, Gothenburg, Sweden). Sperm motility was captured at a rate of 60 frames per second to evaluate velocity. At least 300 sperm and three fields were evaluated in each sample to ensure statistical significance. The Tox Ivos sperm analyzer system reports the median values for each kinematic parameter based on the data collected from each sample; moreover, data in Figure 6 are presented as mean ± SEM.

### 4.10. Quantification of the Sperm Population [Ca^2+^]i in the Presence of CQ, K67, and Rapamycin

Sperm suspensions were preloaded for 30 min with 1 μM Fura-2 AM and 0.05% pluronic acid F-127 simultaneously. Then, the sperm suspensions were centrifuged (6000× *g* for 6 min) at room temperature to remove the excess of Fura-2 AM and pluronic acid F-127, after which they were resuspended in fresh capacitation Tyrode’s medium. The prepared sperm suspensions were transferred into a 96-well plate, and CQ 25 μM, K67 2.5 μM, and rapamycin 250 nM were added. Following 90 min of incubation under capacitation conditions, fluorescence was evaluated using a Synergy 2 Multi-Function Microplate Reader (Bio-Tek Instruments, Winooski, VT, USA) at an alternating wavelength of 340–380 nm and an emission wavelength of 510 nm. As a control, voltage-dependent Ca^2+^ channel inhibition was induced using 1 mM NiCl_2_. Data were interpolated on an activity calibration curve, which was constructed using the same sperm concentration (8–10 × 10^6^ spermatozoa/mL) and incubated in capacitation Tyrode’s medium, containing Ca^2+^ concentrations from 0 to 1000 nM [47].

### 4.11. Quantification of the Sperm Population pH in the Presence of CQ, K67, and Rapamycin

Sperm suspensions were preloaded with the intracellular pH (pHi) fluorescence indicator, BCECF-AM, for 30 min at 37 °C. Then, excess BCECF-AM was removed by centrifugation (6000× *g* for 6 min) at room temperature, and the sperm suspensions were resuspended in fresh capacitation Tyrode’s medium. Subsequently, the sperm suspensions were transferred to a 96-well plate, and CQ 25 μM, K67 2.5 μM, and rapamycin 250 nM were added. After a 90 min incubation under either capacitation or non-capacitation conditions, fluorescence intensity was measured using a Synergy 2 Multi-Function Microplate Reader (Bio-Tek Instruments, Winooski, VT, USA) at an excitation wavelength of 490 nm and an emission wavelength of 535 nm. As a negative control, 1 mM NH_4_Cl_2_ was used. A pH calibration curve was generated using the same sperm concentration (8–10 × 10^6^ spermatozoa/mL) to standardize the data obtained. The sperm suspensions were then incubated in either capacitation or non-capacitation Tyrode’s medium at pH levels ranging from 7.0 to 8.0. Before recording, the sperm were permeabilized using 0.1 Triton X-100 [48].

### 4.12. Evaluation of Sperm Mitochondrial Membrane Potential in the Presence of CQ, K67, and Rapamycin

Sperm mitochondrial membrane potential was evaluated in the presence of CQ 25 μM, K67 2.5 μM, and rapamycin 250 nM. After a 120 min incubation under capacitation conditions, samples were incubated in the presence of mitochondrial membrane potential indicator JC-1 (Molecular Probes, Eugene, OR, USA) for 15 min at 37 °C in the dark. Then, the sperm suspension was washed with PBS to remove excess JC-1. To evaluate the sperm mitochondrial membrane potential, we used a Synergy 2 Multi-Function Microplate Reader (Bio-Tek Instruments, Winooski, VT, USA) with an excitation wavelength of 488 nm and 538 nm of emission for monomers and 596 nm for oligomers. The ratio between JC-1 monomers and oligomers was determined. Carbonyl cyanide m-chlorophenyl hydrazone (CCCP) 50 μM was used as a negative control to inhibit the mitochondrial membrane potential [49,50].

### 4.13. Evaluation of Mouse Sperm AR Induction in the Presence of CQ, K67, and Rapamycin

Sperm suspensions were incubated in Tyrode medium for 2 h at 37 °C. Then, 10 µM calcium ionophore A23187 was added to the samples, immediately followed by CQ 25 μM, K67 2.5 μM, and rapamycin 250 nM, and the incubation was continued for 30 min. After the incubation, the sperm suspensions were centrifuged at 200× *g* for 10 min, and the supernatants were maintained on ice before the AP activity was determined. 4-methylumbelliferyl phosphate was used as a substrate at a concentration of 0.03 mg/mL in a 0.05 M citrate buffer, pH 4.5. After a 30 min incubation at 37 °C, the reaction was stopped by adding 0.4 M glycine buffer, pH 10.4. To measure the AP activity, we used a Synergy 2 Multi-Function Microplate Reader (Bio-Tek Instruments, Winooski, VT, USA) with an excitation wavelength of 360 nm and 449 nm of emission [51].

### 4.14. Statistical Analysis

All experiments were performed at least in triplicate, and graphs were generated using GraphPad Prism 10 (GraphPad Software). For immunofluorescence assays, data analysis was performed using Student’s t-test from three independent replicates (*n* = 3 experimental replicates from pooled samples from 5 mice per condition), where at least 50 spermatozoa per condition in each replicate were analyzed. Western blotting analysis experiments from Figure 1, Figure 2 and Figure 3 was analyzed using Student’s *t*-test from three independent replicates (*n* = 3 experimental replicates from pooled samples from 10 mice per condition). For functional sperm parameters analysis, homogeneity of variances was assessed by Brown–Forsythe test followed by one-way ANOVA test analysis and post hoc Dunnett’s test (*n* = 3 experimental replicates from pooled samples from 5 mice per condition). In all cases, statistical differences were considered significant at *p* < 0.05.

## 5. Conclusions

**Figure 8:**
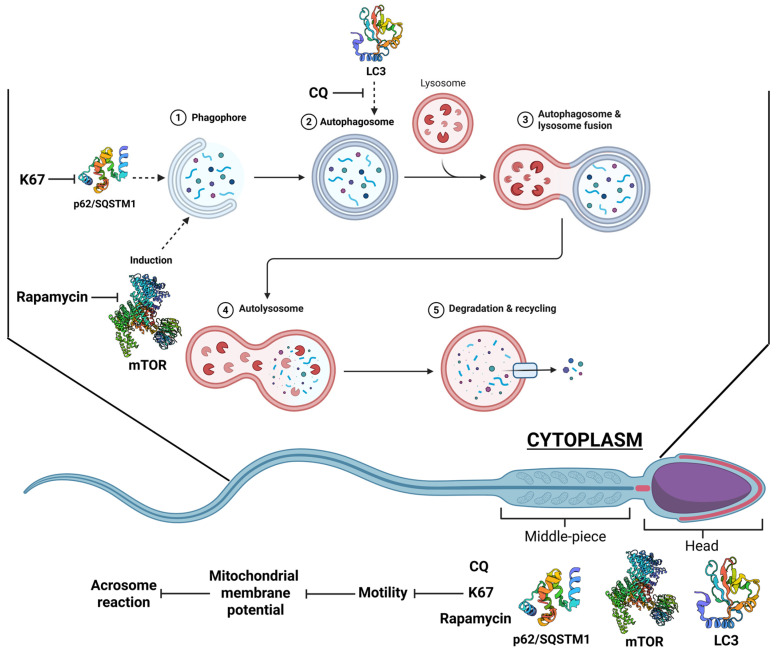
Autophagy-related proteins (LC3, p62/SQSTM1, and mTOR) are present in mouse epididymal sperm. The pharmacological inhibition of these proteins with CQ, K67, and rapamycin did not affect sperm viability or reduce total and progressive motility; however, except for CQ, which decreased pHi, none of the drugs affected capacitation-related events (pHi and [Ca^2+^]i). AR induction was impeded by all the drugs; therefore, we argue that capacitation conditions support the first steps (autophagosome formation) but not further steps of the autophagy process.

Overall, the evidence shown in this work suggests the presence and re-localization of autophagy-related proteins (LC3, p62/SQSTM1, and mTOR) in mouse epididymal sperm during capacitation, which indicates that these cells can undergo at least the initial steps of the autophagy process. Moreover, our results lead us to hypothesize that capacitation conditions can induce the autophagy process without progression to later stages; therefore, we need more experiments (autophagy flux-markers, evaluate phosphorylated forms of p62/SQSTM1 and mTOR, and transmission electron microscopy) to prove this hypothesis. On the other hand, the re-localization of LC3 and p62/SQSTM1 towards the acrosome suggests an active role of this lysosome-related organelle derived from the Golgi apparatus in the autophagy process. Also, treatment of spermatozoa with autophagy inhibitors—CQ, K67, and rapamycin—indicates that autophagy process dysregulation influences functional sperm parameters, such as motility and the acrosome reaction (Figure 8); however, this result could be induced by a non-specific drug effect. Even so, more evidence is required to confirm if capacitation conditions are suitable to induce and maintain the progression of autophagy and the molecules that must be present for full induction.

## Data Availability

The data obtained in this study are available from the corresponding authors upon reasonable request.

## Supplementary Materials

The following supporting information can be downloaded at: https://www.mdpi.com/article/10.3390/ijms262411895/s1.

## Abbreviations

The following abbreviations are used in this manuscript:

LC3	Microtubule-associated protein 1A/1B-light chain 3
p62/SQSTM1	Sequestosome 1
mTOR	Mammalian target of rapamycin
pHi	Intracellular pH
CQ	Chloroquine
AR	Acrosome reaction
[Ca^2+^]i	Intracellular calcium concentration
FDA	Fluorescein diacetate
PI	Propidium iodide
NC	Non-capacitation
Cap	Capacitation

## References

[1] Uribe P., Meriño J., Matus C.E., Schulz M., Zambrano F., Villegas J.V., Conejeros I., Taubert A., Hermosilla C., Sánchez R. (2022). Autophagy is activated in human spermatozoa subjected to oxidative stress and its inhibition impairs sperm quality and promotes cell death. Hum. Reprod..

[2] Yan Q., Zhang Y., Wang Q., Yuan L. (2022). Autophagy: A Double-Edged Sword in Male Reproduction. Int. J. Mol. Sci..

[3] Liu S., Yao S., Yang H., Liu S., Wang Y. (2023). Autophagy: Regulator of cell death. Cell Death Dis..

[4] Kirat D., Alahwany A.M., Arisha A.H., Abdelkhalek A., Miyasho T. (2023). Role of Macroautophagy in Mammalian Male Reproductive Physiology. Cells.

[5] Sharma P., Kaushal N., Saleth L.R., Ghavami S., Dhingra S., Kaur P. (2023). Oxidative stress-induced apoptosis and autophagy: Balancing the contrary forces in spermatogenesis. Biochim. Biophys. Acta Mol. Basis Dis..

[6] Aparicio I.M., Espino J., Bejarano I., Gallardo-Soler A., Campo M.L., Salido G.M., Pariente J.A., Peña F.J., Tapia J.A. (2016). Autophagy-related proteins are functionally active in human spermatozoa and may be involved in the regulation of cell survival and motility. Sci. Rep..

[7] Aparicio I.M., Rojo-Domínguez P., Castillejo-Rufo A., Peña F.J., Tapia J.A. (2023). The Autophagy Marker LC3 Is Processed during the Sperm Capacitation and the Acrosome Reaction and Translocates to the Acrosome Where It Colocalizes with the Acrosomal Membranes in Horse Spermatozoa. Int. J. Mol. Sci..

[8] Song W.-H., Yi Y.-J., Sutovsky M., Meyers S., Sutovsky P. (2016). Autophagy and ubiquitin–proteasome system contribute to sperm mitophagy after mammalian fertilization. Proc. Natl. Acad. Sci. USA.

[9] Correia B., Sousa M.I., Ramalho-Santos J. (2020). The mTOR pathway in reproduction: From gonadal function to developmental coordination. Reproduction.

[10] Moreira B.P., Oliveira P.F., Alves M.G. (2019). Molecular Mechanisms Controlled by mTOR in Male Reproductive System. Int. J. Mol. Sci..

[11] Bai S., Cheng L., Zhang Y., Zhu C., Zhu Z., Zhu R., Cheng C.Y., Ye L., Zheng K. (2018). A germline-specific role for the mTORC2 component Rictor in maintaining spermatogonial differentiation and intercellular adhesion in mouse testis. Mol. Hum. Reprod..

[12] Berezhnov A.V., Soutar M.P., Fedotova E.I., Frolova M.S., Plun-Favreau H., Zinchenko V.P., Abramov A.Y. (2016). Intracellular pH Modulates Autophagy and Mitophagy. J. Biol. Chem..

[13] Kwon D.H., Kim L., Song H.K. (2019). pH-dependent regulation of SQSTM1/p62 during autophagy. Autophagy.

[14] Kazyken D., Lentz S.I., Wadley M., Fingar D.C. (2023). Alkaline intracellular pH (pHi) increases PI3K activity to promote mTORC1 and mTORC2 signaling and function during growth factor limitation. J. Biol. Chem..

[15] Louwagie E.J., Quinn G.F.L., Pond K.L., Hansen K.A. (2023). Male contraception: Narrative review of ongoing research. Basic. Clin. Androl..

[16] Bjørkøy G., Lamark T., Pankiv S., Øvervatn A., Brech A., Johansen T. (2009). Monitoring autophagic degradation of p62/SQSTM1. Methods Enzym..

[17] Fujioka Y., Noda N.N. (2025). Mechanisms of autophagosome formation. Proc. Jpn. Acad. Ser. B.

[18] Khawar M.B., Gao H., Li W. (2019). Mechanism of Acrosome Biogenesis in Mammals. Front. Cell Dev. Biol..

[19] Rogov V., Dötsch V., Johansen T., Kirkin V. (2014). Interactions between autophagy receptors and ubiquitin-like proteins form the molecular basis for selective autophagy. Mol. Cell.

[20] Lee Y.K., Lee J.A. (2016). Role of the mammalian ATG8/LC3 family in autophagy: Differential and compensatory roles in the spatiotemporal regulation of autophagy. BMB Rep..

[21] Hirata S., Hoshi K., Shoda T., Mabuchi T. (2002). Spermatozoon and mitochondrial DNA. Reprod. Med. Biol..

[22] Runwal G., Stamatakou E., Siddiqi F.H., Puri C., Zhu Y., Rubinsztein D.C. (2019). LC3-positive structures are prominent in autophagy-deficient cells. Sci. Rep..

[23] Balestrini P.A., Jabloñski M., Schiavi-Ehrenhaus L.J., Marín-Briggiler C.I., Sánchez-Cárdenas C., Darszon A., Krapf D., Buffone M.G. (2020). Seeing is believing: Current methods to observe sperm acrosomal exocytosis in real time. Mol. Reprod. Dev..

[24] Chakraborty S., Saha S. (2022). Understanding sperm motility mechanisms and the implication of sperm surface molecules in promoting motility. Middle East Fertil. Soc. J..

[25] Berkamp S., Mostafavi S., Sachse C. (2021). Structure and function of p62/SQSTM1 in the emerging framework of phase separation. FEBS J..

[26] Oliveira P.F., Cheng C.Y., Alves M.G. (2017). Emerging Role for Mammalian Target of Rapamycin in Male Fertility. Trends Endocrinol. Metab..

[27] Wang L., Klionsky D.J., Shen H.M. (2023). The emerging mechanisms and functions of microautophagy. Nat. Rev. Mol. Cell Biol..

[28] Tanida I., Ueno T., Kominami E. (2004). LC3 conjugation system in mammalian autophagy. Int. J. Biochem. Cell Biol..

[29] Mauthe M., Orhon I., Rocchi C., Zhou X., Luhr M., Hijlkema K.J., Coppes R.P., Engedal N., Mari M., Reggiori F. (2018). Chloroquine inhibits autophagic flux by decreasing autophagosome-lysosome fusion. Autophagy.

[30] Zhang X.J., Chen S., Huang K.X., Le W.D. (2013). Why should autophagic flux be assessed?. Acta Pharmacol. Sin..

[31] Hargreaves C.A., Rogers S., Hills F., Rahman F., Howell R.J., Homa S.T. (1998). Effects of co-trimoxazole, erythromycin, amoxycillin, tetracycline and chloroquine on sperm function in vitro. Hum. Reprod..

[32] Xia M.C., Cai L., Zhang S., Zhang X. (2018). A cell-penetrating ratiometric probe for simultaneous measurement of lysosomal and cytosolic pH change. Talanta.

[33] Halcrow P.W., Geiger J.D., Chen X. (2021). Overcoming Chemoresistance: Altering pH of Cellular Compartments by Chloroquine and Hydroxychloroquine. Front. Cell Dev. Biol..

[34] Seydi E., Hassani M.K., Naderpour S., Arjmand A., Pourahmad J. (2023). Cardiotoxicity of chloroquine and hydroxychloroquine through mitochondrial pathway. BMC Pharmacol. Toxicol..

[35] Saito T., Ichimura Y., Taguchi K., Suzuki T., Mizushima T., Takagi K., Hirose Y., Nagahashi M., Iso T., Fukutomi T. (2016). p62/Sqstm1 promotes malignancy of HCV-positive hepatocellular carcinoma through Nrf2-dependent metabolic reprogramming. Nat. Commun..

[36] Yasuda D., Yoshida I., Imamura R., Katagishi D., Takahashi K., Kojima H., Okabe T., Ichimura Y., Komatsu M., Mashino T. (2022). Development of p62-Keap1 protein-protein interaction inhibitors as doxorubicin-sensitizers against non-small cell lung cancer. Results Chem..

[37] Wang H., Yang F., Ye J., Dai X., Liao H., Xing C., Jiang Z., Peng C., Gao F., Cao H. (2024). Ginkgo biloba extract alleviates deltamethrin-induced testicular injury by upregulating SKP2 and inhibiting Beclin1-independent autophagy. Phytomedicine.

[38] Poon A., Saini H., Sethi S., O’Sullivan G.A., Plun-Favreau H., Wray S., Dawson L.A., McCarthy J.M. (2021). The role of SQSTM1 (p62) in mitochondrial function and clearance in human cortical neurons. Stem Cell Rep..

[39] Lamming D.W. (2016). Inhibition of the Mechanistic Target of Rapamycin (mTOR)-Rapamycin and Beyond. Cold Spring Harb. Perspect. Med..

[40] Marafie S.K., Al-Mulla F., Abubaker J. (2024). mTOR: Its Critical Role in Metabolic Diseases, Cancer, and the Aging Process. Int. J. Mol. Sci..

[41] Zhu Z., Yue Q., Xie J., Zhang S., He W., Bai S., Tian S., Zhang Y., Xiong M., Sun Z. (2019). Rapamycin-mediated mTOR inhibition impairs silencing of sex chromosomes and the pachytene piRNA pathway in the mouse testis. Aging.

[42] Borbolis F., Ploumi C., Palikaras K. (2025). Calcium-mediated regulation of mitophagy: Implications in neurodegenerative diseases. npj Metab. Health Dis..

[43] Ohne Y., Takahara T., Maeda T. (2009). Evaluation of mTOR function by a gain-of-function approach. Cell Cycle.

[44] Zimmerman M.A., Wilkison S., Qi Q., Chen G., Li P.A. (2020). Mitochondrial dysfunction contributes to Rapamycin-induced apoptosis of Human Glioblastoma Cells—A synergistic effect with Temozolomide. Int. J. Med. Sci..

[45] Hidalgo D.M., Romarowski A., Gervasi M.G., Navarrete F., Balbach M., Salicioni A.M., Levin L.R., Buck J., Visconti P.E. (2020). Capacitation increases glucose consumption in murine sperm. Mol. Reprod. Dev..

[46] Rodríguez-Páez L., Aguirre-Alvarado C., Oviedo N., Alcántara-Farfán V., Lara-Ramírez E.E., Jimenez-Gutierrez G.E., Cordero-Martínez J. (2021). Polyamines Influence Mouse Sperm Channels Activity. Int. J. Mol. Sci..

[47] Li K., Xue Y., Chen A., Jiang Y., Xie H., Shi Q., Zhang S., Ni Y. (2014). Heat shock protein 90 has roles in intracellular calcium homeostasis, protein tyrosine phosphorylation regulation, and progesterone-responsive sperm function in human sperm. PLoS ONE.

[48] Naz R.K. (2014). The Effect of Curcumin on Intracellular pH (pHi), Membrane Hyperpolarization and Sperm Motility. J. Reprod. Infertil..

[49] Harshkova D., Zielińska E., Aksmann A. (2019). Optimization of a microplate reader method for the analysis of changes in mitochondrial membrane potential in Chlamydomonas reinhardtii cells using the fluorochrome JC-1. J. Appl. Phycol..

[50] Carrageta D.F., Freire-Brito L., Oliveira P.F., Alves M.G. (2022). Evaluation of Human Spermatozoa Mitochondrial Membrane Potential Using the JC-1 Dye. Curr. Protoc..

[51] Pietrobon E.O., Domínguez L.A., Vincenti A.E., Burgos M.H., Fornés M.W. (2001). Detection of the Mouse Acrosome Reaction by Acid Phosphatase. Comparison With Chlortetracycline and Electron Microscopy. J. Androl..

