# Supplementary figures and images for: Autophagy-Related Proteins Influence Mouse Epididymal Sperm Motility
Source: Int J Mol Sci. 2025 Dec 10;26(24):11895. doi: 10.3390/ijms262411895 (PMC12733172; doi:10.3390/ijms262411895)

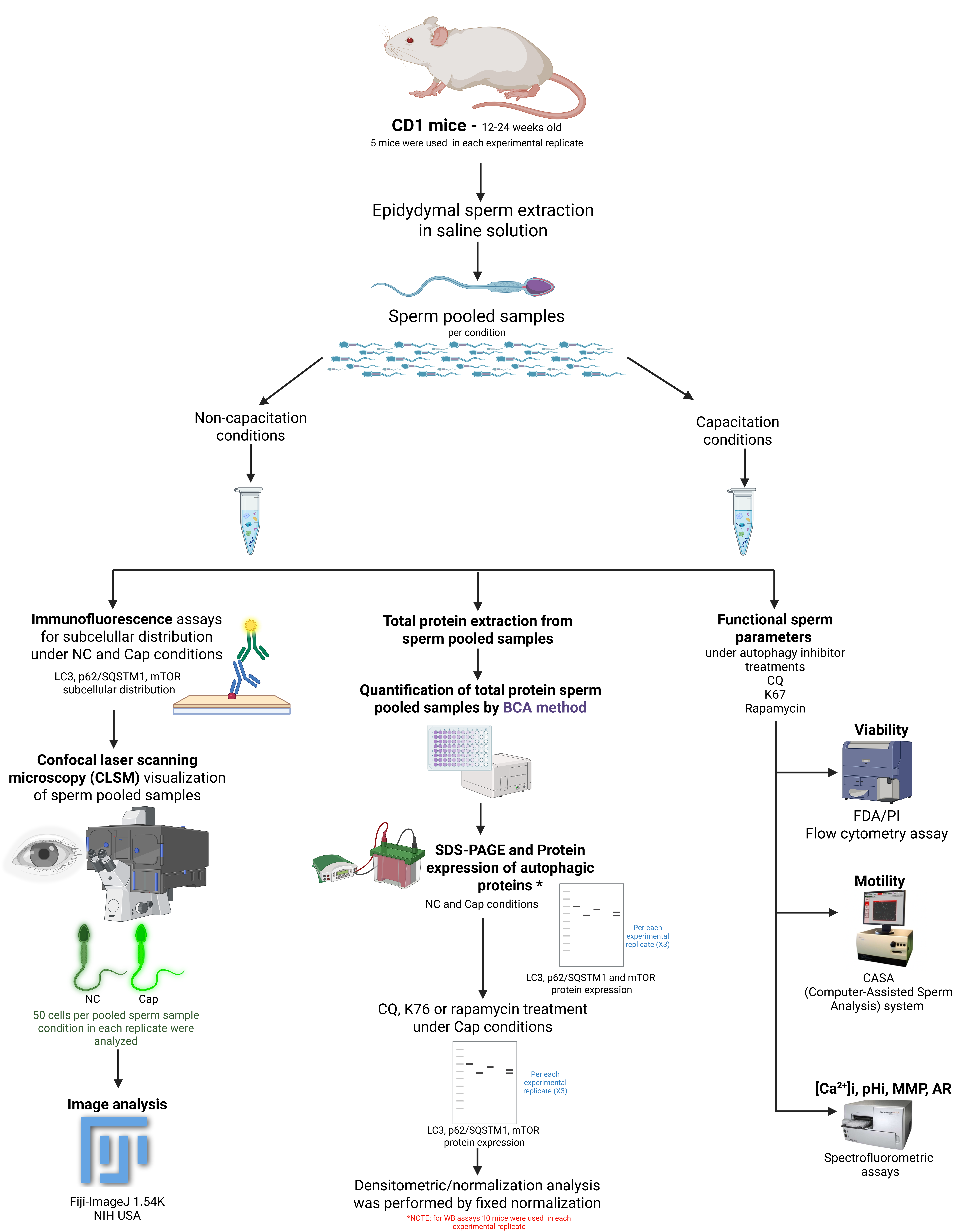

Supplement: Supplementary file 1 [file ijms-26-11895-s001.zip › ijms-3919945-supplementary.png]
